# Accelerated reconstruction of rat calvaria bone defect using 3D-printed scaffolds coated with hydroxyapatite/bioglass

**DOI:** 10.1038/s41598-023-38146-1

**Published:** 2023-07-27

**Authors:** Nasrin Fazeli, Ehsan Arefian, Shiva Irani, Abdolreza Ardeshirylajimi, Ehsan Seyedjafari

**Affiliations:** 1 Department of Biology, Science and Research Branch, Islamic Azad University, Tehran, Iran; 2 Department of Microbiology, School of Biology, College of Science, University of Tehran, Tehran, Iran; 3 Urogenital Stem Cell Research Center, Shahid Beheshti University of Medical Sciences, Tehran, Iran; 4 Department of Biotechnology, College of Science, University of Tehran, P.O.Box: 141556455, Tehran, Iran

**Keywords:** Biotechnology, Cell biology, Developmental biology, Molecular biology

## Abstract

Self-healing and autologous bone graft of calvaraial defects can be challenging. Therefore, the fabrication of scaffolds for its rapid and effective repair is a promising field of research. This paper provided a comparative study on the ability of Three-dimensional (3D) printed polycaprolactone (PCL) scaffolds and PCL-modified with the hydroxyapatite (HA) and bioglasses (BG) bioceramics scaffolds in newly bone formed in calvaria defect area. The studied 3D-printed PCL scaffolds were fabricated by fused deposition layer-by-layer modeling. After the evaluation of cell adhesion on the surface of the scaffolds, they were implanted into a rat calvarial defect model. The rats were divided into four groups with scaffold graft including PCL, PCL/HA, PCL/BG, and PCL/HA/BG and a non-explant control group. The capacity of the 3D-printed scaffolds in calvarial bone regeneration was investigated using micro computed tomography scan, histological and immunohistochemistry analyses. Lastly, the expression levels of several bone related genes as well as the expression of miR-20a and miR-17-5p as positive regulators and miR-125a as a negative regulator in osteogenesis pathways were also investigated. The results of this comparative study have showed that PCL scaffolds with HA and BG bioceramics have a great range of potential applications in the field of calvaria defect treatment.

## Introduction

The most usual type of injury is bone fracture that can be caused by ageing, metabolic failures, accidents, or trauma^1^. Bone has the ability to regenerate and repair itself in small injuries^2,3^. But large bone fractures require transplantation, which poses significant clinical challenge^4^. Bone regeneration via matrix implantation into the affected area is an alternative approach^5^. Another suitable method for the treatment of bone fractures, osteoporosis, and bone abnormalities is bone tissue engineering, which combines engineered materials, biomedical technology and renewable stem cells^6^. Engineered scaffolds significantly affects mass transport and supports cell proliferation, adhesion and growth^7^. Controlled biodegradability, suitable mechanical strength and interconnected pore structure with the desired pore size and porosity for cell growth are the characteristics of an ideal scaffold^8,9^. An appropriate bone scaffold should have an interconnected porous system with porosity of about 65% and pore size of approximately 200–800 µm, in order to mimic the porous structure of a natural bone^10^. There is a great interest in the development of construction methods to raise the functionality of scaffolds. Three-dimensional (3D) printing technology has been widely used in the field of biomedical tissue regeneration engineering, which utilizes computer-aided design (CAD) software to construct complex 3D structures^11–13^. With the advancement in medical technology, surgeons have been able to scan patients' skull defects by computed tomography (CT) scanner and prepare a 3D scaffold model based on digital data using biomaterials to help reconstruct the skull deformities^14–18^. One of the most commonly and cost-effective of 3D printing technologies is the fused deposition modeling (FDM) technique, which can be used to fabricate scaffolds by injecting the biomaterials layer by layer from a temperature-controlled nozzle^17^. An example of biomaterials is Poly-ε-caprolactone (PCL), a biocompatible polymer, that has been approved by the Food and Drug Administration (FDA). But PCL is not suitable for cell attachment and proliferation due to its surface hydrophobicity^19^. Therefore, the use of this polymer can be limited in the construction of tissue engineering scaffolds. To solve this issue, bioactive ceramics such as hydroxyapatite (HA) and bioactive glasses (BG), which are similar to the bone mineral phase, are used to improve cell attachment of PCL scaffolds. Bioactive ceramic scaffolds can result in strong chemical bonds with the bone tissue, due to the formation of HA-like layers of bones^20^. Therefore, bioactive ceramics are the most widely used materials in bone tissue engineering due to their high potential in bonding with bone and their stimulating effect on new bone formation^21^. HA (Ca_5_(PO_4_)_3_(OH)) is a natural mineral component of bone and shows excellent bioactivity, biocompatibility, bioconductivity, non-toxicity and non-inflammatory properties. It is very hard but it’s fragile and its degradation rate inside the body is very slow, and for this reason, it is used together with natural or synthetic polymers to make scaffolds^22^. HA is beneficial for bone formation because it stimulates growth factors such as bone morphogenetic proteins (BMPs)^23^. BG is one of the most promising bioceramics with good biocompatibility in vitro and in vivo and after being placed in biological fluid, BG produce bioactive HA layers, which binds to biological tissues and improves bone formation. The disadvantages of BG are its low strength and brittleness^24^.microRNAs (miRNAs) are a class of endogenous, evolutionarily conserved, single-stranded RNAs approximately 21–23 nucleotides in length acting as post-transcriptional regulators by targeting 3′untranslated regions (UTR) of target mRNAs to coordinate a wide range of biological prosses^25^. In recent years, the relationship between miRNAs and bone formation has attracted much attention. Studies have shown that miRNAs have a regulatory effect on osteoblastic differentiation and bone development^26^.

In this research, we used a FDM 3D printing technology and an easy post-fabrication modification method to prepared scaffolds that can effectively promote the repair of calvaria in rat calvarial defect model. Four groups of scaffolds including PCL, PCL/HA, PCL/BG, and PCL/HA/BG were used in vivo experiments after ensuring of adhesion and proliferation of human adipose derived mesenchymal stem cells (hADMSCs) on the surface of the scaffolds.

The novelties of this paper are as follows:

- This research has performed a comprehensive comparative study of surface modification of 3D printed PCL scaffolds using HA and BG bioceramics alone and in combination in repair of damaged calvarial bone in rats. The rate of new bone formation was evaluated using micro CT scan, histological analysis including: Alizarin red, Hematoxylin–eosin (H&E), Masson’s trichrome staining and immunohistochemical (IHC) assays. Furthermore, bone-related genes expression in mRNA level was evaluated.
- The effect of nanotomography of PCL, PCL/HA, PCL/BG and PCL/HA/BG 3D printed scaffolds in the expression of microRNAs and the target genes involved in rats calvarial bone regeneration has been investigated.

## Materials and methods

In the following reported experiments on live vertebrates and methods used, all relevant guidelines and regulations, particularly ARRIVE guidelines^27^, have been fully considered and noticed. Furthermore, the experimental methods were approved by Iran National Committee for Ethics Biomedical Research (IR.IAU.SRB.REC.1401.344).

### Materials

PCL (Mn = 80,000) was purchased from Sigma-Aldrich, USA. HA and BG were purchased from NikCeram Razi, Iran.

### 3D printing and post-fabrication modification

The porous 3D printing scaffolds were fabricated by a 3D printer (Omid Afarinan Mohandesi Ayande, BioFabX2, Iran) and FDM method using software (Repetier Host V2.1.3, 2011_2018). PCL pellets melt into a heating cylinder and leave a nozzle with a lay-down pattern of 0/90°. Circular four-layer scaffolds with a diameter of 6 mm and the same parameters: 0.5 mm diameter nozzle, a layer thickness of 0.2 mm, a distance of 0.3 mm between the two strings, a temperature of 110 °C, and a speed of 2 mm/s were fabricated for in vitro and in vivo assays. To improve the hydrophilicity of the surface of 3D-printed scaffolds, plasma treatment was done using a low-frequency plasma generator at 90 GHz, with a cylindrical quartz reactor (Diener Electronics, Nagold, Germany). Pure oxygen gas was conducted into the reaction at 0.4 mbar pressure, followed by ignition of glow discharge for 3 min. A 1% (w/v) solution of HA, BG and HA/BG (1% each) was prepared and to better dispersion of microparticles, the solutions were put in an ultrasonic bath for 20 min at 37 °C. Plasma-treated scaffolds were immersed in HA, BG, and HA/BG solutions to cover microparticles on the surface of scaffolds overnight. Then they were washed with deionized water and dried. Four groups of the scaffolds (PCL, PCL/HA, PCL/BG, PCL/HA/BG) were sterilized by UV and ethanol and used for several tests.

### Scaffolds characterization

To characterize surface morphology of PCL, PCL/HA, PCL/BG and PCL/HA/BG scaffolds, a field emission scanning electron microscope (FeSEM, Mira3, Tescan, Czech Republic) was used to image the samples according to our previously reported method^20^.

### Cell morphology and adhesion

To evaluate the ability of 3D-printed scaffolds in cell adhesion, 10^4^ cells/cm^2^ of hAMSCs were seeded on the scaffolds and TCPs (tissue culture polystyrene) as a control. After 7, 14 days of incubation, seeded scaffolds were examined in term of cell attachment using FeSEM (FEI ESEM Quanta 200) according to our previously reported method^20^.

### Animal model

In this study, 10-week-old male wistar rats weighing between 250 and 300 g from the University of Tehran, Faculty of Pharmacy (Ethics Committee) were used. The rats were kept in suitable environmental conditions in terms of light, temperature and nutrition.

### Scaffold implantation

**Figure 1:**
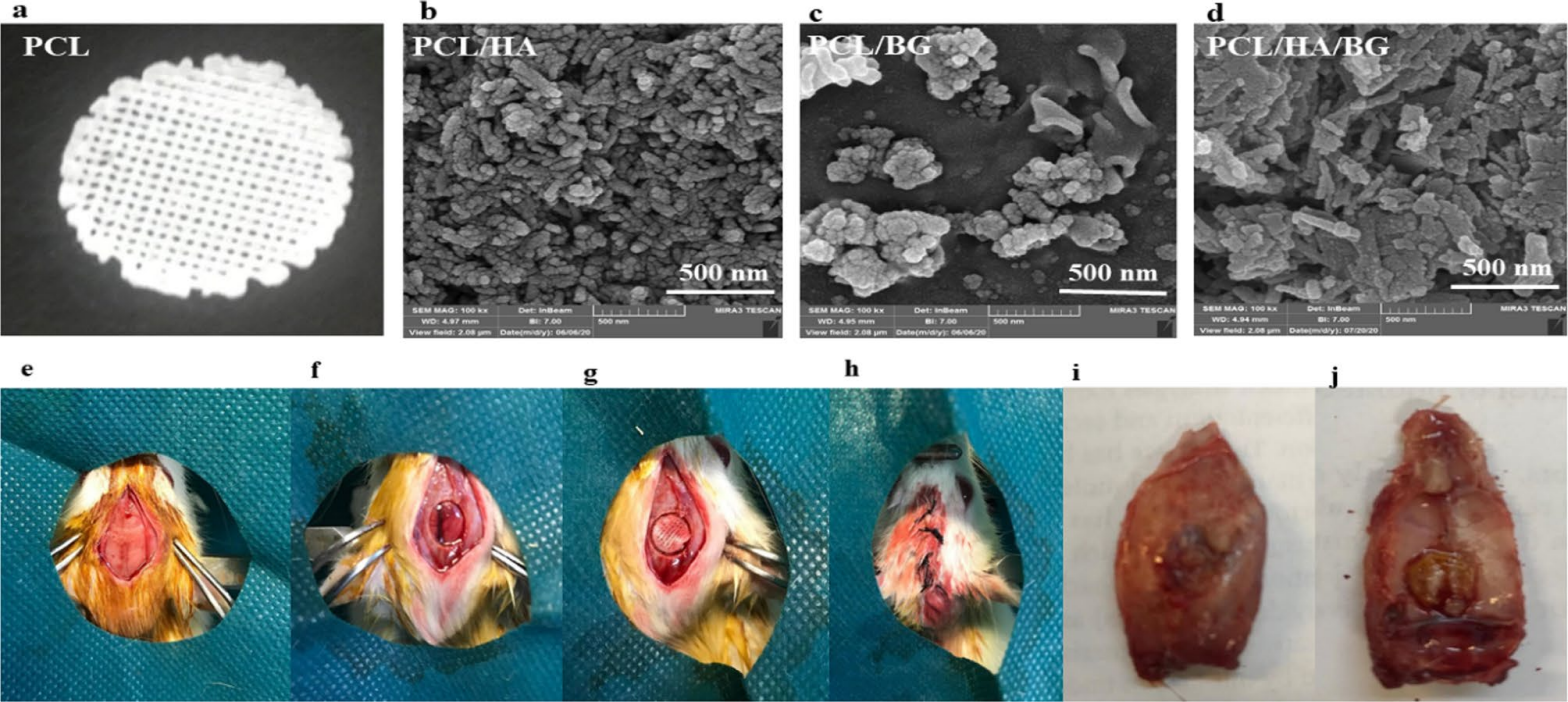
(**a**) The macroscopy of morphology of PCL scaffold and the nanbioceramics on the surface. (**b**) The surface morphology of PCL/HA, (**c**) The surface morphology of PCL/BG and (**d**) The surface morphology of PCL/HA/BG scaffolds at 100 KX magnifications. (**e**)–(**h**) Stages of creating a lesion in rat calvaria and implantation. (**i**) Reconstruction of the calvaria defect in control group after 30 days. (**j**) Reconstruction of the calvaria defect in groups with 3D printed scaffolds graft after 30 days.

After the surgery of rats (see Supporting Information 1), the 3D-printed scaffolds were implanted in calvaria area. Four groups were researched: (1) Rats with the bone defect without implantation (control) (2) rats with the bone defect filled with PCL scaffolds (3) rats with the bone defect filled with PCL/HA scaffolds (4) rats with the bone defect filled with PCL/BG scaffolds (5) rats with the bone defect filled with PCL/HA/BG scaffold. For each group were anesthetized (with an intraperitoneal injection 5 mg/kg of xylazine and 100 mg/kg of ketamine) 30 days after the surgery and their heads were cut off using a guillotine with a sharp blade and the transplanted parts were separated. Samples for histological analysis involved IHC, H&E, masson’s trichrome, alizarin red staining and micro CT scan were placed in 10% formalin and to evaluate the expression of genes and microRNAs involved in bone differentiation by Real-time PCR were placed in − 70 °C freezer (Fig. 1e–j).

### In vivo osteogenesis of the scaffold

The tissue samples were first embedded in paraffin, then deparaffinized for staining (see Supporting Information 1).

#### H&E staining

H&E staining is a common histological staining that results in the cell nucleus turning blue, keratin, elastic fibers, and fibrin bright red. Slides were put in Hematoxylin (H9627-Sigma) for 7 s, and after washing they were put in Li_2_CO_3_ (1.05680-Sigma) for 2 s and then 3 min in eosin (HT110116-Sigma). For dehydration, the specimens were placed in ethanol 90% and 100% for 4 s each. At the end to clarify the tissues, they were placed in xylol and observed with optical microscope.

#### Alizarin red staining

Deparaffinized slides are put in the alizarin (1.06278-Merck) for 1 min. To remove the excess colors, the slides were placed in acetone/xylol solution (1:1), after being in acetone 100%, and then observed with optical microscope.

#### Masson’s trichrome staining

First, slides were dehydrated with decreasing degrees of ethanol. Then, they were placed in Boen's solution in a 56 °C autoclave for an hour and afterwards washed. Next, Hematoxylin vagrant dye was poured on the tissue for 10 min. The samples were then washed again and placed in Biebrich scarlet-acid fuchsin solution for 10 min. After 10 min of washing, phosphotungstic-phosphomolybdic acid solution was poured on the slides. Finally, they were placed in aniline blue solution for 20 min. After washing with water, the slides were put in 1% acetic acid (1.00056-Merck), and after dehydrating and using xylol, they were observed.

#### IHC staining

The slides were located in a TBS 1X (T5912-Sigma) solution inside the microwave oven until the point of boiling, then washed with PBS. Triton (T8787-Sigma) 0.3% was used for 30 min to permeabilize the cells. The slides were washed with PBS, and then goat serum (G9023-Sigma) 10% was added to the samples for 45 min. Primary antibody (SC-365797, SC-33645) (1:100 with PBS) was added to the samples and refrigerated for 24 h. Specimen were washed several times with PBS, while secondary antibody (ORB688924) was added to the samples (1:150) and placed in a dark incubator at 37 °C for one and a half hours. Finally, after washing the slides, DAPI (D9542—Sigma) dye was added in a dark room and after 20 min, the samples were washed and observed with a fluorescent microscope (Olympus).

#### Micro-CT scan

In this study, we used an in vivo X-ray micro-CT scanner (LOTUS inVi-vo, Behin Negareh Co., Tehran, Iran) at the Preclinical Core Facility (TPCF) based at the Tehran University of Medical Sciences. LOTUS-in Vivo has a cone beam micro-focus X-ray source and a flat panel detector. In order to obtain the best possible image quality, the X-ray tube voltage and its current were set to 50 kV and 150 μA, respectively, and frame exposure time set to 2 s by 1.4 magnification level. Total scan duration was 49 min. Slice thicknesses of reconstructed images were set to 47 µm. All the protocol settings process was controlled by LOTUS-in Vivo-ACQ software. The acquired 3D data was reconstructed using LOTUS inVivo-REC by a standard Feldkamp, Davis, Kress (FDK) algorithm. The ImageJ and Avizo softwares were used to analyze the images^28,29^.

In order to calculate bone regeneration volume for the samples, the region of interest (ROI) was filtered by median filter (3 × 3) to remove noise, and contrast enhancement was applied to improve the visual appearance of the images. Then, the images were segmented using segmentation algorithms into:

1. A material for control group; bone regeneration that represented with white.
2. Four materials for treatment groups; bone regeneration, scaffold, nanoparticles, and polymer that each represented with dark gray, white, light gray, and gray respectively.
3. Three materials for polymer group; bone regeneration, scaffold, and polymer that each represented with dark gray, white, and light gray respectively

Finally, the ratio of the bone regeneration volume to the total volume was calculated (%).

$${\text{Total Volume}}\left( {\text{treatment groups}} \right) = {\text{Volume of cavity created}} - ({\text{Scaffold volume}} + {\text{Nano}} - {\text{particles volume}} + {\text{Polymer volume)}}.$$

$${\text{Total Volume}}\left( {\text{control group}} \right) = {\text{Volume of cavity created }}\left( {{\text{Diameter}} = {6}\;{\text{mm}},{\text{ Depth}}/{\text{Height}} = {1}\;{\text{mm}}} \right)$$

In this experiment, the volume bone regeneration in the groups of control, PCL, PCL/HA and PCL/BG was calculated.

#### Gene expression

Evaluation of rat bone-specific genes including *ALP*, *BMPR2*, *Osteocalcin*, *Runx2*, *COL1*, *Smad7* (inhibitor) at the mRNA level and a number of microRNAs involved in bone differentiation including miR-20a, miR-125a, miR-17-5p using Real-time PCR was performed. This technique requires three steps: 1. RNA extraction 2. Synthesis of cDNA 3. qPCR technique.

*Step 1—RNA extraction* First, the samples were manually homogenized in a porcelain flask. To obtain the cell lysate, Trizol (1 ml) (Kiazist, Iran) was added to the microtubes of the samples, then 200 ml of chloroform (DRM. CHEM, Iran) was poured on the samples and manually shaken for about 20 s. After incubating for 15 min at room temperature, they were centrifuged for 15 min in 4 °C and 12,000 RPM (Hettich, Germany). The clear phase in the upper part of the microtube containing RNA was transferred to another microtube. In the microtubes containing the upper phase (containing RNA), 500 ml of isopropanol (DRM. CHEM, Iran) was added and they were shaken several times to mix the contents. The microtubes were then placed in a -20 °C freezer for a short period of 5 to 10 min. To precipitate of RNA, the samples were centrifuged for 10 min at 4 °C and 12,000 RPM. The supernatant solution was drained and the sediment were washed and vortexed with 200 ml of ethanol. After that, the samples were centrifuged at 7500 RPM and 4 °C. Finally, the supernatant was discharged and the microtube was placed upside down on a paper towel for 15 min to evaporate the alcohol. In order to dissolve the RNA sediment, 20 ml of DEPC or RNase free water (Thermo, USA) was added to the microtubes. The extracted RNA were then stored in a freezer at − 80 °C.

*Step 2—Synthesis of cDNA* After thawing the extracted RNA, they were placed on ice and vortexed for a while. To make cDNA, mixture of 5 µl of RT mix (including RT buffer, RT enzyme, random hexamer primer), 1 µl of oligodt primer and 1 µl of DEPC water and mix 1 µl of RNA to this volume of 9 µl in microtubes (0.2 ml) has been Added (Easy cDNA Synthesis Kit, Iran). They were placed in the thermocycler (KIAGENE, Iran) according to the following temperature program (Table 1).

**Table 1:** RT program.

RT-PCR	25 °C	10 min	1X
	47 °C	60 min	
	85 °C	5 min	
Cooling	4 °C	2 min	1X

*Step 3—qPCR technique* First, we took out all the materials needed for Real-time PCR from the freezer and after vortexing, we put them on ice. Then, according to the Table 2, we prepared a mixture of different PCR components for each gen, and after mixing, 9 μl was poured into each microtube and 1 μl of the cDNA was added to each vial (the final volume of each vial is 10 μl). After mixing and vortexing, the prepared microtubes containing the PCR reaction were placed in the device (Real-time PCR thermocycler, ABI Stepone, USA) according to the executive program of Table 3. In the end, the datas including CT numbers (threshold cycle), the melting curve of each gene is ready for analysis. The relative expression of genes and MicroRNAs was calculated using 2^−ΔΔCT^. The sequences of the primers are presented in Table 4, and U6 was used as an internal control.

**Table 2:** Materials for real time PCR program.

SYBR green master mix (addbio, Korea)	5 μl
Forward primer (10 μM) (Pishgam)	0.5 μl
Reverse primer (10 μM) (Pishgam)	0.5 μl
H2O	3 μl
Total volume	9 μl + 1 μl cDNA

**Table 3:** Real time PCR programs.

Step	Temperature	Time	Cycle
Initial denaturation	95 °C	5 min	1X
Amplification	95 °C	15 s	40X
	60 °C	15–20 s	
	72 °C	15–30 s	
Melting curve	95 °C	15 s	1X
	65 °C	1 min	
	65–95 °C, slope: 0.3 °C/s		
Cooling	30 °C	20 s	1X

**Table 4:** Sequences of primer pairs used for quantitative real-time PCR.

Name	Sequence
miR-20a-RT	GTC GTA TGC AGA GCA GGG TCC GAG GTA TTC GCA CTG CAT ACG ACC TAC CT
miR-20a-F	CAT GCC TAA AGT GCT TAT AGT G
miR-125aRT	GTC GTA TGC AGA GCA GGG TCC GAG GTA TTC GCA CTG CAT ACG ACT CAC AG
miR-125a-F	TCCCTGAGACCCTTTAAC
miR-17-5p-RT	GTC GTA TGC AGA GCA GGG TCC GAG GTA TTC GCA CTG CAT ACG AC C TAC CT
Mir-17-5p-F	TGA CAA AGT GCT TAC AGT GC
SMAD7-F	CCTTAGCCGACTCTGCGAACTA
SMAD7-R	CCAGATAATTCGTTCCCCCTGT
BMPR2-F	AGAGACCCAAGTTCCCAGAAGC
BMPR2-R	CCTTTCCTCAGCACACTGTGCA
GAPDH-F	AGGTCGGTGTGAACGGATTTG
GAPDH-R	TGTAGACCATGTAGTTGAGGTCA
Collagen I (Col-I)-F	TGGAGCAAGAGGCGAGAG
Collagen I (Col-I)-R	CACCAGCATCACCCTTAGC
Runx2-F	GCCTTCAAGGTGGTAGCCC
Runx2-R	CGTTACCCGCCATGACAGTA

### Statistical analysis

In this experiment, 30 rats were selected and divided into five groups: four groups with scaffold implantation and one group without implantation (n = 6). The results were analyzed with GraphPad software. One-way analysis of variance (ANOVA) was selected to compare the results. A *P*-value < 0.05 was considered as the level of significance. In all of the graphs, the letters a, b, c and d are used to show significant differences between the groups.

### Ethical approval

The experimental methods were approved by Iran National Committee for Ethics Biomedical Research (IR.IAU.SRB.REC.1401.344).

## Results

### Cell adhesion on 3D-printed scaffolds

**Figure 2:**
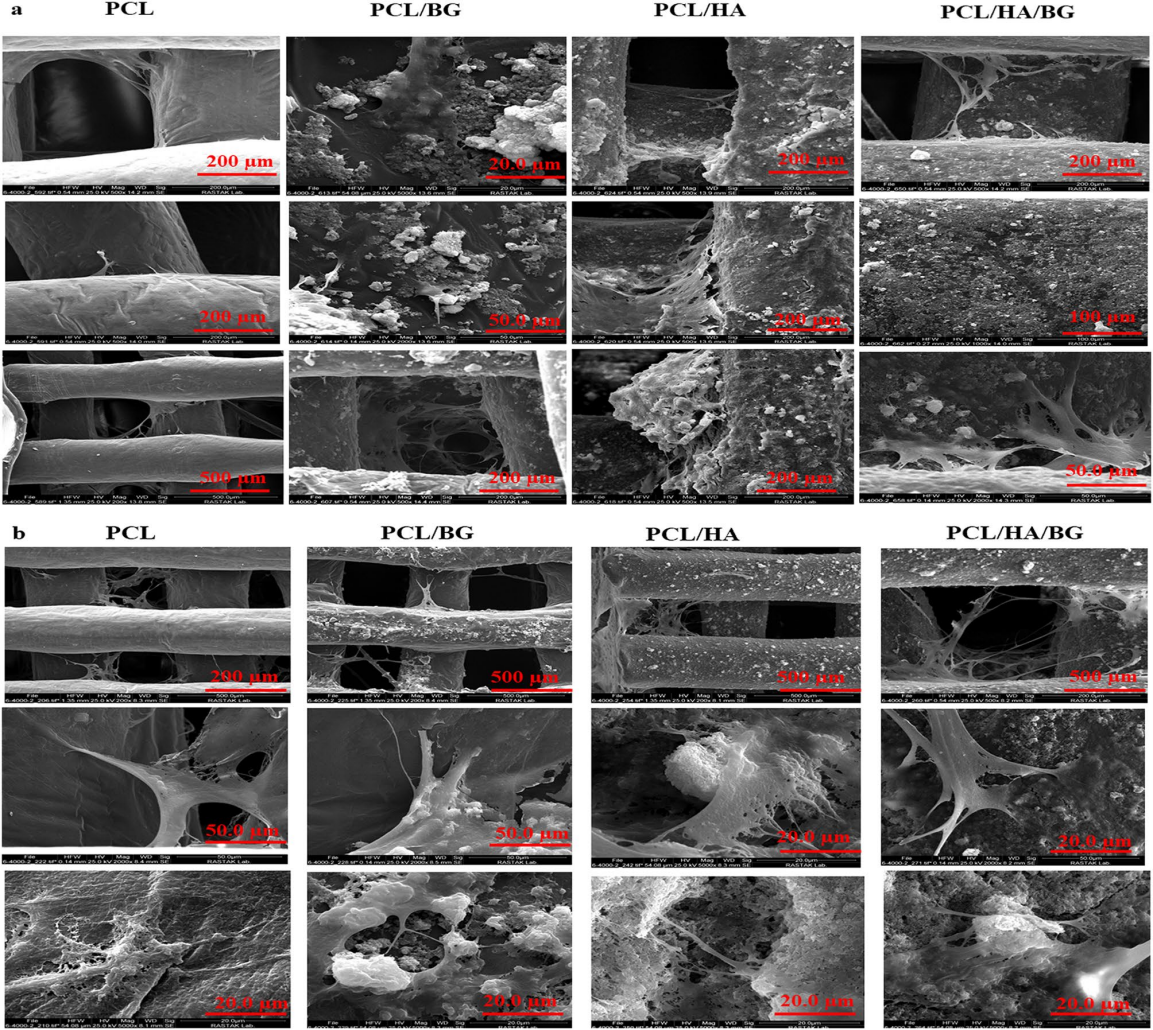
(**a**) FeSEM images of hADSCs cultured on the PCL, PCL/HA, PCL/BG, PCL/HA/BG scaffolds on days 7. (**b**) FeSEM images representing hADSCs cultured on the PCL, PCL/HA, PCL/BG, PCL/HA/BG scaffolds on days 14.

After treatment the scaffolds with nanoparticles, to ensure proper coating of scaffolds surface with bioceramics, the surface morphologies of the PCL/HA, PCL/BG, and PCL/HA/BG scaffolds were characterized by FeSEM. As seen from the micrographs (Fig. 1b–d), At high magnification, bioceramic particles can be seen on the surface of scaffolds and the surface of scaffolds is well covered by nanoparticles. Furthermore, the macroscopy of PCL scaffold has been shown in Fig. 1a. Also, investigating the effect of the surface the scaffolds on cell adhesion and distribution was performed using seeding hAMSCs on PCL, PCL/HA, PCL/BG, and PCL/HA/BG scaffolds at days 7 and 14. FeSEM images showed that the 3D-printed PCL scaffolds and the scaffolds modified with the nanoparticles could serve as suitable substrates for the attachment of hAMSCs (Fig. 2a,b).

### Histological assay

Histological analysis of calvarial defects with and without the implanted scaffolds over the period of one month after surgery were performed and the results are given in the following subsections.

#### H&E staining

**Figure 3:**
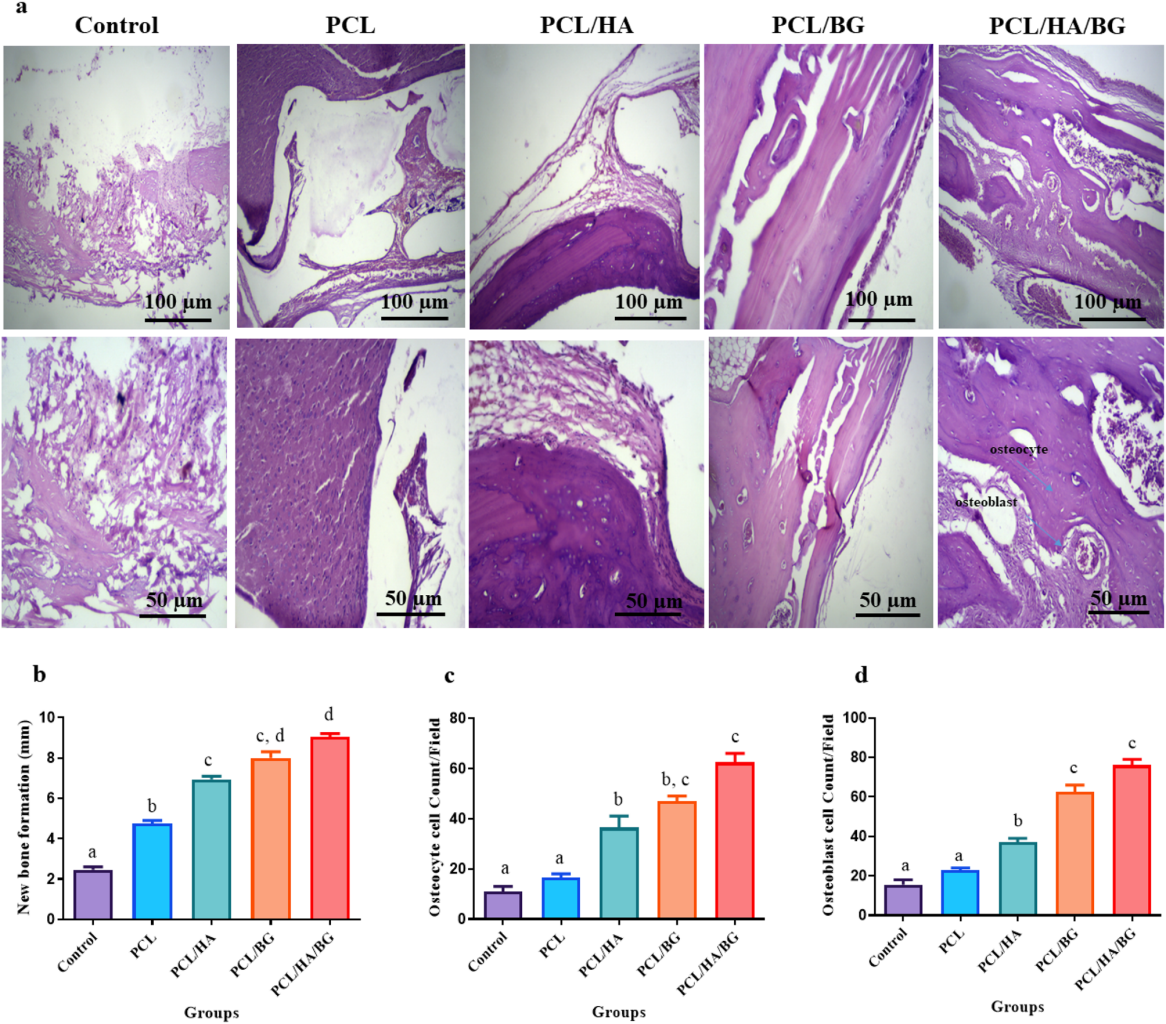
3D printed PCL/HA/BG scaffolds accelerated rat calvarial bone defect regeneration. (**a**) Microscopic appearance of tissue section after H&E staining 30 days post-operation. (**b**) Diagram of new bone formation, (**c**) counting of osteoblasts (**d**): counting of osteocyte in PCL, PCL/HA, PCL/BG, PCL/HA/BG groups.

New bone formation is seen in all groups, but rats that are implanted with scaffolds, especially PCL/nanobioceramics scaffolds showed better ossification. Groups PCL/HA/BG show the best results in terms of new bone formation, followed by the groups PCL/BG and PCL/HA, while not showing a significant difference in this matter. In the samples stained with H&E, counting of osteoblast and osteocyte was also carried out. From the point of view of the number of these cells, there was no significant difference between the two control and PCL groups, but the three groups PCL/HA/BG, PCL/HA and PCL/BG had more osteocyte and osteoblast cells, and in this respect, they showed a significant difference with the control group. The highest number of bone cells was counted in the PCL/HA/BG group (Fig. 3a,d).

#### Alizarin red staining

**Figure 4:**
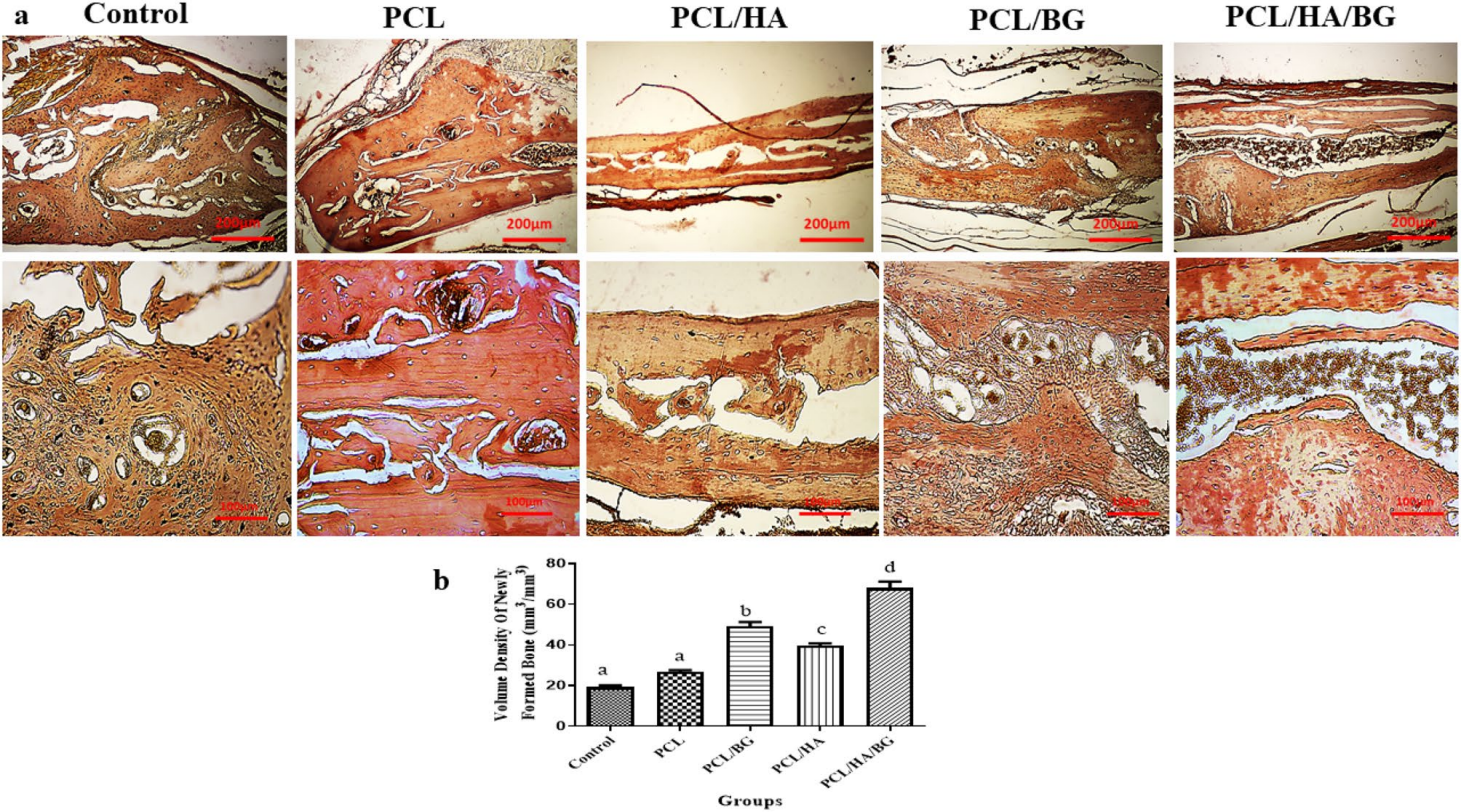
(**a**) Microscopic appearance of tissue section after alizarin red staining 30 days post-operation. (**b**) Diagram of alizarin red staining of tissue section 30 days after implantation in PCL, PCL/HA, PCL/BG, PCL/HA/BG groups.

Alizarin red staining was done to observe the amount of mineralization of the tissue formed at a certain time. Areas of new bone formation were evaluated using Image J software. As shown in Fig. 4a,b, the highest amount of mineralization was related to the PCL/HA/BG scaffold, so that there is a significant difference even with the two groups PCL/HA and PCL/BG. The Scaffolds coated with nanoparticles showed a significant difference compared to the PCL group and the control group in terms of bone formation, while no significant difference was seen between the control and PCL groups.

#### Masson’s trichrome staining

**Figure 5:**
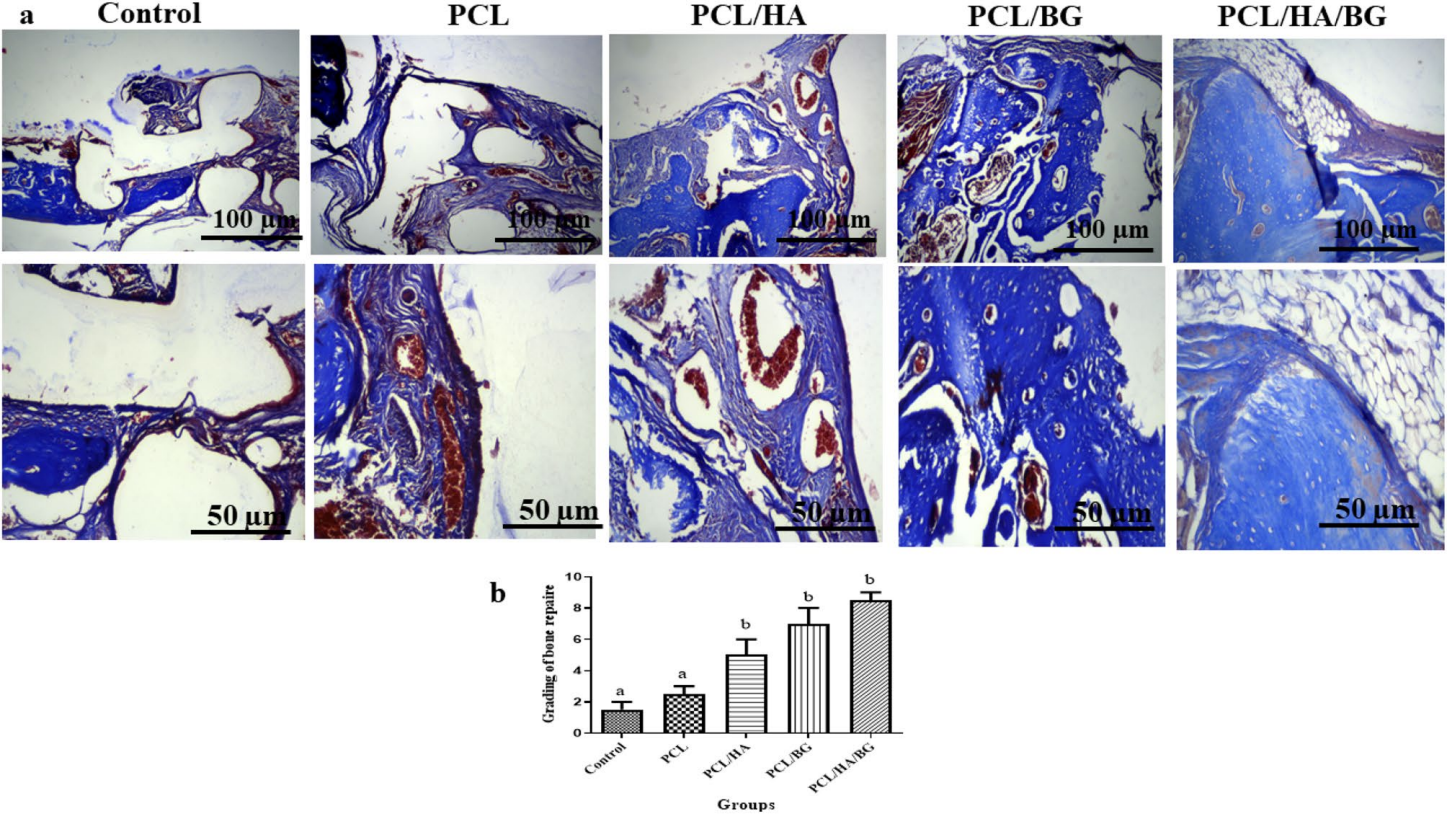
Bone regeneration evaluated by masson’s trichrome staining. (**a**) Masson’s trichrome staining was performed to show the collagen deposition and newly formed bone within the implanted region at 30 days post-surgery. (**b**) Diagram of masson’s trichrome staining in PCL, PCL/HA, PCL/BG, PCL/HA/BG groups.

To evaluate the bone regeneration, masson’s trichrome staining was also performed (Fig. 5a,b), to show the newly formed bone and collagen deposition within the implanted constructs at one month post-implantation. Masson trichrome staining data further revealed that the PCL/HA/BG group showed the best bone integration and the increased new bone regions in PCL/HA and PCL/BG groups compare to control and PCL group. The images obtained from the staining of the samples were analyzed in terms of grading of bone repair based on the research work of Maliki Gerji^30^. The data showed that mature bone was produced only in the PCL/HA/BG group in a small amount and immature bone formation took place in the PCL/BG and PCL/HA groups.

#### IHC staining

**Figure 6:**
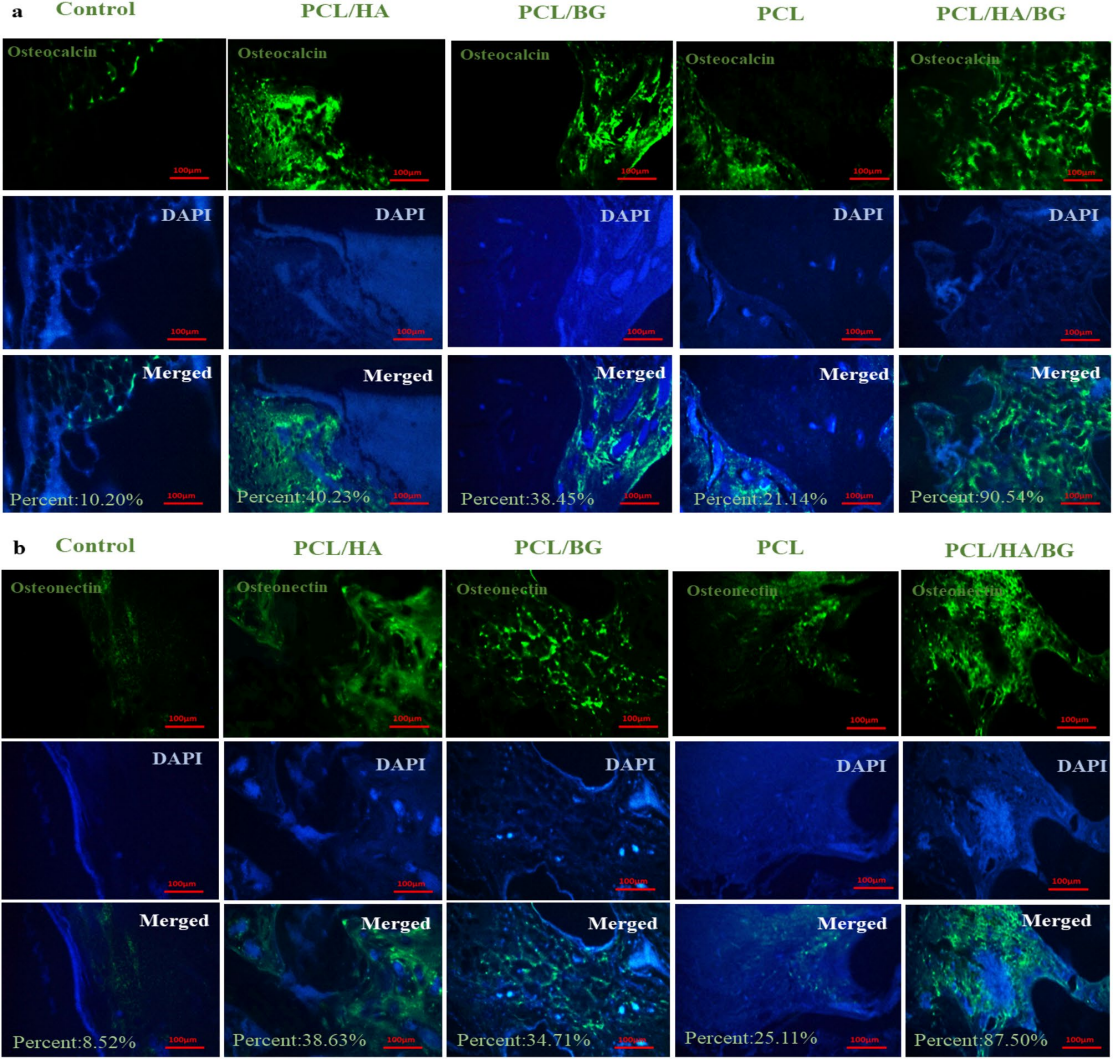
Immunohistochemistry staning of tissue implanted section 30 days post-operation in PCL, PCL/HA, PCL/BG, PCL/HA/BG groups. (**a**) The amount of osteocalcin protein in implanted tissue section. (**b**) The amount of osteonectin protein in implanted tissue section.

Osteocalcin and osteonectin are markers of bone tissues. In order to investigate the level of calvarial bone regeneration in different groups of rats, the specific expression of two proteins, osteocalcin and osteonectin, was analyzed IHC method. As shown in Fig. 6a,b, the highest amount of osteocalcin and osteonectin proteins was observed in the PCL/HA/BG groups. PCL/HA and PCL/BG groups were came second in terms of two these proteins presence. The amount of osteonectin and osteocalcin was very low in the rats that had damage in the calvaria area without implantation.

### Micro-CT scan

**Figure 7:**
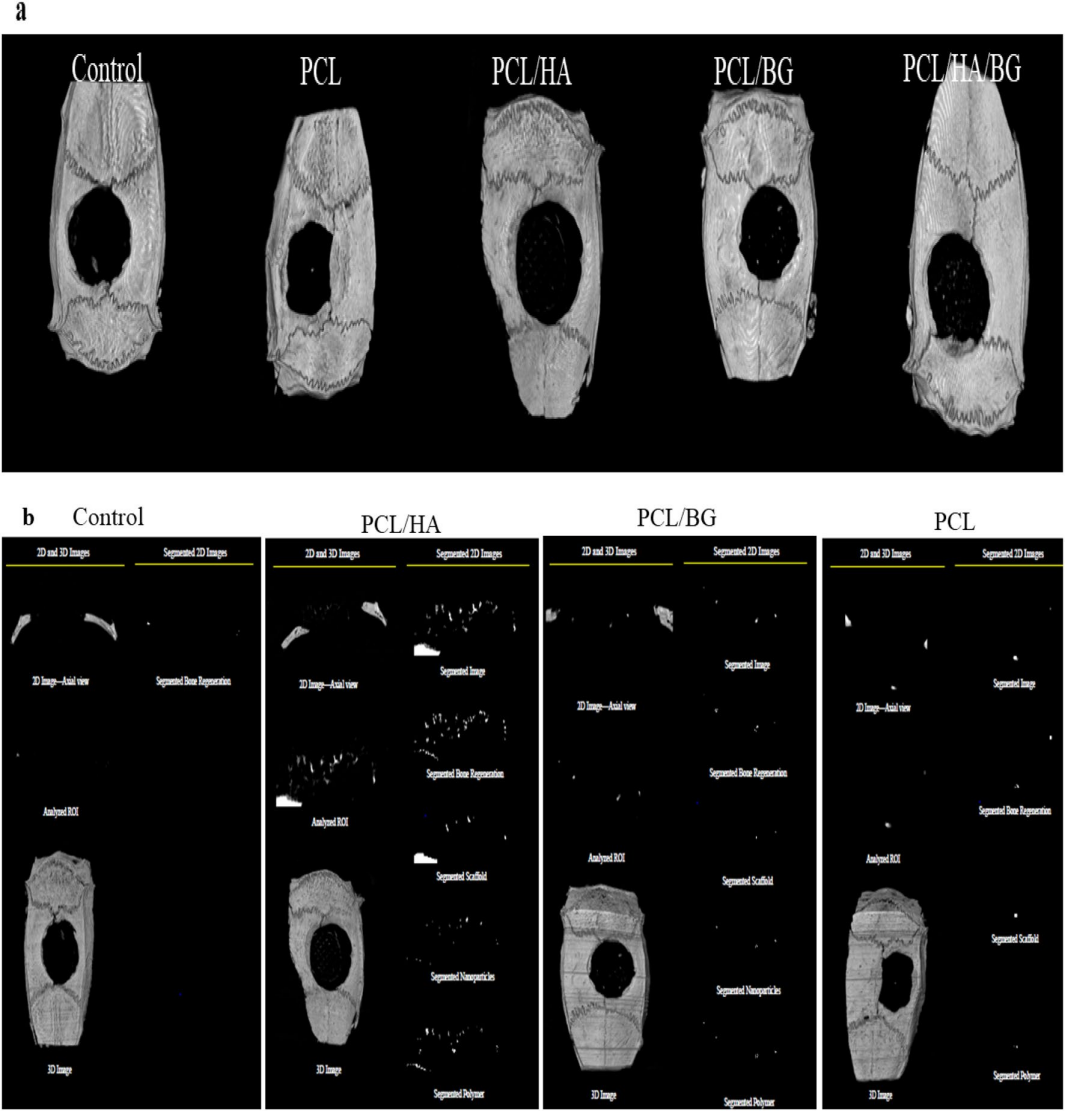
The analyzed 2D-μCT and 3D-μCT images of calvarial bone regeneration took place in the rats that received the PCL, PCL/HA, PCL/BG, PCL/HA/BG scaffolds and control group without implantation. (**a**) 3D-μCT images, (**b**) 2D-μCT (The ImageJ and Avizo softwares were used to analyze the figures).

The images were obtained from the micro-CT scan showed that the best calvarial bone regeneration took place in the rats that received the PCL scaffold with HA nanoparticles. The rats that were implanted by the PCL/BG scaffolds also had some bone repair, but the control rats and the rats that received the PCL scaffold without nanoparticles performed very poorly in terms of calvarial bone regeneration. The analyzed 2D-μCT and 3D-μCT images are also shown in Fig. 7a,b.

The ratio of the bone regeneration volume to the total volume in the PCL/HA group was calculated compared to the control group. The rate of bone regeneration in rats that received PCL/HA scaffolds was 2.5297%, while in rats with the bone defect without implantation (control) was 0. 2517% means more than10 times regeneration. Also this ratio in PCL and PCL/BG groups was 0.1057 and 0.1480 respectivly.

### Gene expression

#### Bone related genes expression

**Figure 8:**
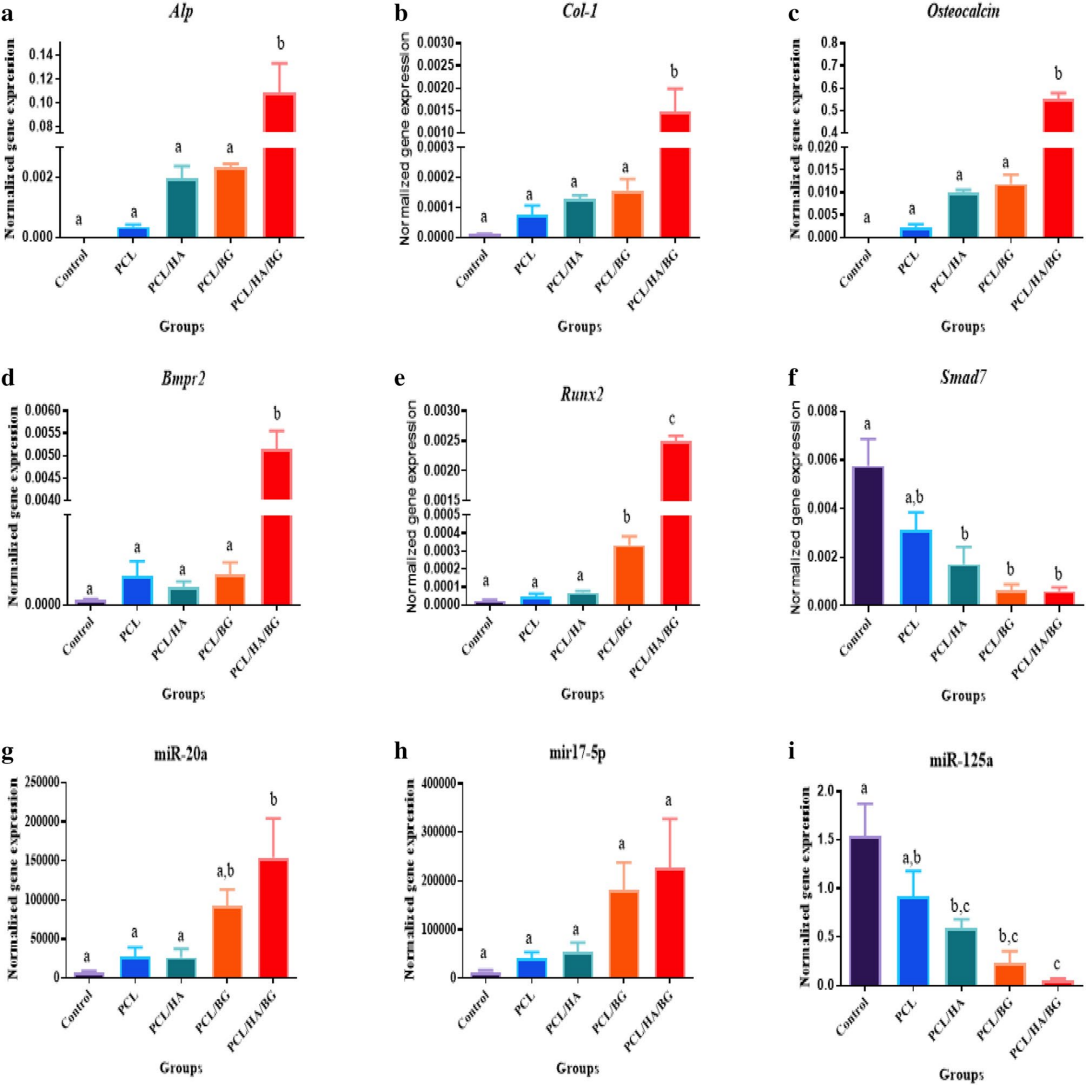
Evaluation of relative expression rat bone-specific genes in mRNA level and MicroRNAs expression in tissue implanted region in PCL, PCL/HA, PCL/BG, PCL/HA/BG groups after 30 days post-operation. (**a**) ALP, (**b**) COL1, (**c**) Osteocalcin, (**d**) BMPR2, (**e**) Runx2, (**f**) Smad7, (**g**) miR-20a, (**h**) miR-17-5p, (**i**) miR-125a.

To evaluate the expression level of osteoblast markers at the mRNA level, Real-time PCR was performed. The relative expression of *ALP*, *Col1*, *Osteocalcin*, *BMPR2* and *Runx2* genes was measured to investigate bone differentiation and calvarial bone regeneration. The highest expression of *ALP*, *Col1*, *Osteocalcin* genes was seen in the PCL/HA/BG groups, so that the expression of these genes in the PCL/HA/BG groups was significantly different compare to the PCL, PCL/HA, PCL/BG groups and the control group. It should be noted that this difference was less seen in *COL1* gene expression. Also, the expression level of *BMPR2* in PCL/HA/BG groups was significantly higher than other groups. The analysis of *Runx2* gene expression in the four tested groups and the control group showed that the PCL/HA/BG group showed a significance difference with the other groups, the PCL/BG group also showed a difference with the PCL and control groups, while this difference was also seen with PCL/HA group to a lesser extent. *Smad7* gene has an antagonistic and inhibitory role in TGFβ/BMP signaling. In this study the lowest of expression of *smad7* determined in PCL/bioceramics and showed a significance difference with control (Fig. 8a–f).

#### MicroRNAs expression

Figure 8g–i showed that, there are different expressions of miR-20a, miR-17-5p and miR-125a in the four groups with the 3D-printed scaffolds implanted relative to each other as well as to control without implantation. An increasing trend of expression of miR-20a as a positive regulator was clearly observed in the rats that received PCL/HA/BG compared to the control and PCL groups even PCL/HA groups 30 days post-operation. Although there was no significant difference in the expression of miR-17-5p in the experimental groups. MiR-125a, as a negative regulator, was downregulated in osteogenesis pathway. PCL/HA/BG groups showed the lowest expression of miR-125a compare to other groups.

## Discussion

The calvaria has a thin and spherical structure. Autologous bone and allogeneic bone grafts cannot effectively regenerate large-sized calvaria defects. Clinically, calvaria defects need a suitable scaffold for repairing^31^. To solve such problems, metal, inorganic, and polymer materials have been widely developed^32,33^. Considering that the calvaria defect of patients has a personalised shape, 3D printing processing technology brings a new direction for the preparation of personalised scaffolds of calvaria defects based on patient CT data^34^. FDM is one of the most popular 3D printing techniques suitable for fabricating polymer scaffolds that uses heat. The material used in this technique is polymer or a combination of polymer and ceramic. In this method, the desired material is melted by heat and squeezed out of the nozzle and deposited layer by layer and a scaffold is created. The process temperature depends on the melting temperature of its structural material^35^. PCL is one of the most studied polymers and has been proven to support bone cells. PCL exhibits little inflammatory and immunological stimulation, due to its biocompatibility, the FDA has approved the use of PCL.

Bone bioactivity and cellular affinity of synthetic polymers are weak and some degradation problems, mechanical problems and acidic cellular environment have been reported. In the case of PCL, adding BG as a ceramic phase can compensate for its inherent hydrophobic nature and poor cell adhesion, and also improve its mechanical properties^36^. By surface modification of polymer scaffolds with bioceramics like HA, BG, tri-calcium phosphate (TCP), and calcium silicate (CS) as biomaterials based on calcium, phosphate, and silicate, this compound affects the cells and improves osteogenesis^37^. There is a lot of evidence that topography affects the differentiation of stem cells^38–40^. For example, in our previous study, we showed the effect of surface modification of polymer scaffolds with bioceramics in bone differentiation of stem cells^20^. But the effect of topography on bone regeneration in vivo is less clear. In this study, the potential of fabricated 3D printed scaffolds without and with post fabrication modification in new bone formation was evaluated using a calvaria bone defect model in rats. Before in vivo experiments, the potential of PCL, PCL/HA, PCL/BG, and PCL/HA/BG scaffolds in cell attachment evaluated using hAMSCs at days 7 and 14 after cell seeding by FeSEM. SEM analysis revealed that the four groups of the scaffolds served as an excellent surface for the cell attachment. The cells expanded on the scaffolds to form a cell layer and the newly proliferating cells communicated with each other, which were poured into the scaffold’s pores. In PCL-bioceramics scaffolds, the interaction between the polymer, nanoparticles, and the cells was seen and the cell elongation on the nanoparticles was significant. In PCL-bioceramic scaffold, the roughness of surface and the hydrophilicity property of HA and BG improved cell attachmen.

For in vivo experiments, after ensuring that the rats were unconscious, the scalp was removed in the rat calvaria area and right in the center of the calvaria, an area with the diameter of the fabricated scaffolds (6 mm) was removed from the rat calvaria bone, and the 3D printed scaffolds implanted at the site of Calvaria damage^41,42^. After 30 days, five groups of rats including: control (without implantation), PCL, PCL/HA, PCL/BG, PCL/HA/BG, were anesthetized again and their heads were cut and the transplanted part was separated from the rats' heads. Samples for histological analysis involved IHC, H&E, Masson’s trichrome, Alizarin red staining and micro CT scan were placed in 10% formalin and to evaluate the expression of gens and microRNAs involved in bone differentiation by Real-time PCR were placed in − 70 °C.

H&E resulting showed that the rats with scaffold implantation in the injuried area had a significant difference compared to the control group without scaffold implantation in terms of new bone formation and better repair. However, the best group was the PCL/HA/BG. Cell counting of osteoblasts and osteocytes revealed that there was no significant difference between the control and PCL groups, but PCL/HA/BG, PCL/HA and PCL/BG groups had a greater number of osteocyte and osteoblast cells. The highest number of bone cells were counted in the PCL/HA/BG group. Masson's trichrome staining was used to demonstrate new bone formation and collagen deposition in the implanted constructs. The data showed that mature bone was only showed in a small amount in the PCL/HA/BG group and immature bone formation took place in the PCL/BG and PCL/HA groups. Alizarin red staining was performed to evaluate the degree of mineralization of the damaged part of calvaria and compared the results with Masson's trichrome and H&E staining results. The analysis of the results showed a good mineralization rate in PCL/HA/BG scaffolds implanted in the calvaria injury area, so that this group has a significant difference in terms of mineralization rate with the PCl/HA and PCL/BG groups. The PCL and control groups showed the lowest amount and there was no significant difference between the two. Overall, PCL scaffolds coated with the bioceramics showed better results in this test in comparison to the control group and even PCL alone. In fact the peresence of HA and BG bioceramics, improved bone regeneration. In order to investigate the amount of calvarial bone repair in different groups of tested rats, the specific expression of two proteins, osteocalcin and osteonectin, as markers of bone tissues, was investigated by IHC. The highest amounts of osteocalcin and osteonectin proteins were observed in the PCL/HA/BG group. PCL/HA and PCL/BG groups were in the second row in terms of protein presence, and the PCL group without bioceramic nanoparticles was in the third row. The amount of osteonectin and osteocalcin was very low in rats that had defect in the calvaria area without implantation of scaffold. Therefore, the presence of bioceramic particles in the PCL 3D printing scaffold has increased the expression of osteocalcin and osteonectin, as Yong Sang Cho and his group showed in 2019 that the expression of osteonectin and collagen type I in the PCL 3D printing scaffold was lower than PCL/HA composite scaffold^43^. The images obtained from the micro CT scan confirmed the histological analyzes and showed that the best calvarial bone regeneration occurred in the rats that received the PCL/HA/BG scaffold. The rats with the PCL/HA and PCL/BG scaffolds also had some bone repair, but the control and the rats that received the PCL scaffold without nanoparticle coating performed very poorly in terms of calvarial bone regeneration. The ratio of the bone regeneration volume to the total volume in the PCL/HA group was calculated compared to the control group. The rate of bone regeneration in PCL/HA sgroup was 2.5297%, while in control group rats was 0. 2517% means more than10 times regeneration. Xing et al.^44^ demonstrated the topographical effect of 3D printed PCL scaffolds on increasing the proliferation and osteogenic differentiation of urine-derived stem cells for bone regeneration. For this purpose, they prepared PCL scaffolds with a nanotopographic surface of PCL particles and compared it with PCL scaffold alone in vitro. Also, they investigated the effect of these scaffolds in the regeneration of rat skull bone. Their results based on H&E and Masson's trichrome staining tests and micro CT scan revealed that when the PCL scaffold surface was modified with soluble polycaprolactone particles, the skull was repaired better^44^. Xu et al.^45^ evaluated the effect of 3D printed PCL scaffold alone and also with laponite (LAP) in rat calvarial bone regeneration. Twelve weeks after transplantation, this group investigated the rate of new bone formation in the calvarial defect area in rats by using micro CT scan images and histological analyzes such as H&E, trichrome and alizarin red staining. Data and micro-CT images showed that the rate of new bone formation was higher in rats that received PCL/LAP scaffold grafts than in rats that received PCL scaffold grafts^45^. The effect of polyvinyl alcohol/β-tricalcium phosphate/icariin composite scaffolds in the rapid and effective repair of calvaria defects investigated by Xu et al.^46^. Similar to this study, this group created a circular lesion in the middle region of the parietal bone of the calvaria. Micro CT scan images and histological analyzes of H&E and Masson's trichrome staining showed that the rats with defect without scaffold had no significant repair even up to the 12th week, but in the rats with a scaffold implantation, new bones had filled some of the holes in the scaffold^46^. Furthermore, the relative expression of *ALP*, *Col1*, *Osteocalcin*, *BMPR2*, *Runx2* and *smad7* genes was measured by Real Time PCR method to investigate bone differentiation and calvarial bone regeneration. Best expression of bone genes *ALP*, *Col1*, *Osteocalcin* was seen in PCL/HA/BG group, so the expression of these genes in PCL/HA/BG group was significantly different compare to PCL, PCL/HA, PCL/BG and control group. The analysis of *Runx2* gene expression in the four groups and control revealed that three-component PCL/HA/BG group had a significant difference with other groups and the PCL/BG group also showed a significant difference with the PCL and control groups, while this difference was seen to a lesser extent with the PCL/HA group. *Runx2* is a transcription factor involved in bone formation that activates the promoter of bone-specific genes such as *ALP* and *COL1* during the early stages of bone differentiation. Therefore, it is reasonable that the PCL/HA/BG scaffold stimulated the mineralization of the extracellular matrix by synthesizing *COL*1 to form the matrix for osteoid formation which is attributed to the increase of *Runx2* level by HA and BG in PCL/HA/BG scaffold. At the end, the nanotopography effect of 3D-printed scaffolds on microRNAs expression (miR-20a, miR-17-5p, miR-125a) involved in BMP signaling was examined by qPCR. Osteogenic differentiation is a complex process that is regulated by a variety of factors including microRNAs. Studies by Zhang et al. in 2011 showed that the expression of endogenous miR-20a increases during bone differentiation, and the expression of bone markers and regulators *BMP2*, *BMP4*, *Runx2*, *OSX*, *OCN*, and *OPN* increased at the same time, While the expression of adipocyte markers *PPARy* and osteoblast antagonists *Bambi*, *Crim1* is decreased. They showed that miR-20a promotes osteogenic differentiation through upregulation of BMP/Runx2 signaling by silencing *PPARy*, *Bambi* and *Crim1*^47,48^. In this research, the highest level of miR-20a expression was observed in the rats that received the PCL/HA/BG scaffolds, and the highest level of Runx2, OCN genes expression that involved in BMP/Runx2 signaling was also observed in this groups. Smad7 has an inhibitory role in TGF β/BMP signaling and is regulated by several miRs. miR-17-5p directly targets Smad7 in MSCs and promotes osteoblastic differentiation and cell proliferation^26^. In this study, there was no significant difference in the expression of miR-17-5p in the experimental groups but groups with bioceramic coating showed little expression of smad7 as inhibtor. Some researches determined that miR-125a negatively regulates osteoblastic differentiation of hAMSCs by targeting smad4^49,50^. So, we expected to see downregulation of miR-125a in the PCL/HA/BG groups compare to control based on other obtained results. According to the diagram, the downregulation of miR-125a in PCL/HA/BG groups compared to control was shown.

**Figure 9:**
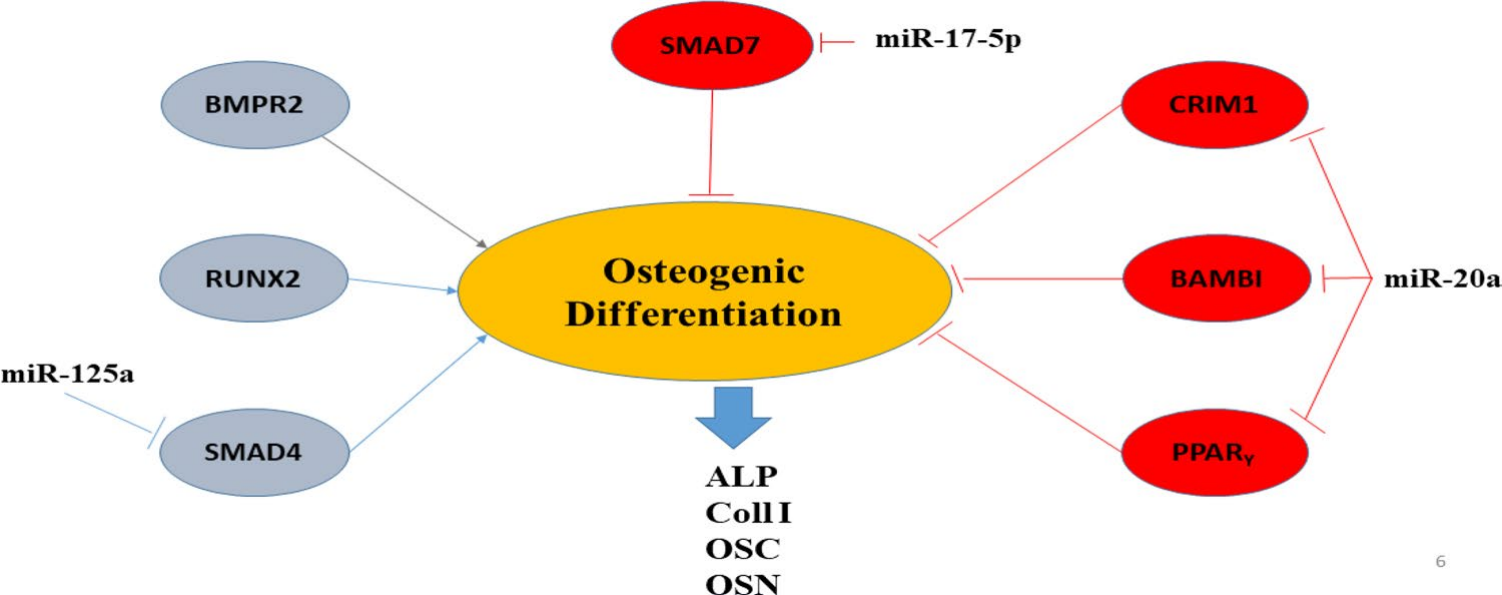
Schematic diagram of the interaction of miR-20a, miR-125a and miR-17-5p in osteogenic differentiation pathways.

The histological staining showed that the PCL/HA/BG scaffolds have been more successful in rats calvaria bone regeneration. HA and BG bioceramics have osteoconductive and osteoinductive properties that lead to the osteointegration between the host bone and the implant. Topographical cues are recognized by cellular sensors such as integrins which result in biochemical signaling cascades and changed the expression of genes and proteins. The rats have implanted with PCL/HA/BG scaffolds, HA/BG nanoparticles stimulated BMP signaling pathway. BMP signaling has a pivotal role in the regulation of bone induction, maintenance, and repair. In the topography of the PCL/HA/BG scaffold, the highest expression levels of miR20a and miR17-5p (as positive regulators in osteogenesis) were observed resulting in increased bone markers including *ALP*, *Col 1*, *Osteocalcin*, *BMPR2*, *RunX2*, by inhibiting *Crim 1*, *Bambi*, *PPAR*_*Y*_ as osteogenesis inhibitors. Also, the PCL/HA/BG scaffold had more reduced the expression of miR-125a level as a negative osteogenesis regulator. Therefore, the presence of both HA and BG bioceramics in rats that received PCL/HA/BG implant, improved BMPs and ALP and resulted in enhancing BMP signaling and more regeneration of defect area (Fig. 9).

The analysis results of in vivo revealed that serious calvaria defect require the use of a suitable scaffold. The PCL/HA/BG scaffold is a suitable scaffold for bone tissue engineering and is recommended for clinical applications.

## Conclusions

Clinically, calvaria defects need a suitable scaffold to regenerate large-sized injuries. 3D printing technology brings a new direction for the preparation of personalised scaffolds of calvaria defects based on patient CT data. The porous PCL scaffolds were fabricated using FDM-3D printing. Post fabrication modification of the scaffolds was performed using HA, BG and HA/BG bioceramics. First, the potential of the scaffolds in attachment of hAMSCs was investigated in vitro. Then the ability of fabricated scaffolds in reconstruction and new bone formation was evaluated using a rat calvaria bone defect model. The results of histological and IHC analysis, micro CT scan and evaluation of genes and MicroRNAs expression involved in bone regeneration indicated that in PCL/HA/BG scaffold, the interaction of HA and BG caused improved calvarial bone regeneration and it could be a suitable for applications in bone tissue engineering.

## Supplementary Information

Supplementary Information.

## Data Availability

The dataset used for analysis in this paper is available from the corresponding author upon request.
