# Supplementary material for: Accelerated reconstruction of rat calvaria bone defect using 3D-printed scaffolds coated with hydroxyapatite/bioglass
Source: Sci Rep. 2023 Jul 27;13:12145. doi: 10.1038/s41598-023-38146-1 (PMC10374909; doi:10.1038/s41598-023-38146-1)
Supplement: Supplementary file 1 — Supplementary Information. [file 41598_2023_38146_MOESM1_ESM.pdf]

Accelerated Reconstruction of Rat Calvaria Bone Defect Using 3D-Printed Scaffolds Coated with Hydroxyapatite/Bioglass

Nasrin Fazeli[1], *Ehsan Arefian*[2], *Shiva Irani*[1], *Abdolreza Ardeshirylajimi*[3], *Ehsan Seyedjafari*[4]*

[1] Department of Biology, Science and Research Branch, Islamic Azad University, Tehran, Iran

[2] Department of Microbiology, School of Biology, College of Science, University of Tehran, Tehran, Iran

[3] Urogenital Stem Cell Research Center, Shahid Beheshti University of Medical Sciences, Tehran, Iran

[4*] Department of Biotechnology, College of Science, University of Tehran, Tehran, Iran.

[*]**Corresponding address:** Ehsan Seyedjafari, Department of Biotechnology, College of Science, University of Tehran, P.O.Box: 141556455, Tehran, Iran. Tel: +98(21) 66412556. Fax: +98-21-66405141. Email: seyedjafari@ut.ac.ir

Surgical procedure and scaffold implantation

The rats were anesthetized with an intraperitoneal injection of xylazine (5 mg/kg) and ketamine (100 mg/kg). After being shaved scalp cranial region and sterilized by betadine and alcohol, the skin were detached to expose the calvaria bone. Then using Trephine (6 mm) a lesion was created in Calvaria and 3D-printed scaffolds were implanted and sutured, followed by an intramuscular injection of enrofloxacin (10 mg/kg).

Paraffin embedding

First, the specimen was removed from the formalin solution, it was placed in 10% nitric acid for a while to soften it and be cut. In order to dehydrate, the tissues were placed in ethanol from low grades to absolute alcohol for 30 min each. To remove the alcohol from the tissue, the samples were placed in xylol for 50 min. Finally, the samples embedded in paraffin and transversal were cut with a microtome to a thickness of 5 μm and placed on slides.

Paraffin melting

First to melt the paraffin, the slides were placed in oven 90°C for 20 min. Second, they were placed in xylol (1330-20-7-Sigma) for 15 min. In order to hydrate, the slides were put in descending order in 100% to 70% ethanol, and finally, in distilled water (in each for 5 minutes).
